# A Dual-Band Multi-Linear Polarization Reconfigurable Antenna for Body-Centric Wireless Communication Systems

**DOI:** 10.3390/s25123630

**Published:** 2025-06-09

**Authors:** Dingzhao Chen, Foxiang Liu, Xuexuan Ruan, Yanhui Liu

**Affiliations:** 1 Yangtze Delta Region Institute (Quzhou), University of Electronic Science and Technology of China (UESTC), Quzhou 324000, China; dingzhaochen@csj.uestc.edu.cn (D.C.); ruanxx@csj.uestc.edu.cn (X.R.); 2 School of Electronic Science and Engineering, University of Electronic Science and Technology of China (UESTC), Chengdu 611731, China; 3 School of Information Engineering, Nanchang University, Nanchang 330029, China; fxliu@ncu.edu.cn

**Keywords:** polarization reconfigurable antenna, monopole antenna, dual-band antenna

## Abstract

A novel dual-band multi-linear polarization reconfigurable (MLPR) antenna for body-centric wireless communication systems (BWCS) is presented in this paper. The design comprises five symmetrically arranged multi-branch radiating units, each integrating an elliptical patch and curved spring branch for the Medical Implant Communication Service (MICS) band (403–405 MHz), and a pair of orthogonal strip patches for the Industrial, Scientific and Medical (ISM) 2.45 GHz band (2.40–2.48 GHz). By selectively biasing PIN diodes between each unit and a central pentagonal feed, five distinct LP states with polarization directions of $0^{\circ }$, $72^{\circ }$, $144^{\circ }$, $216^{\circ }$, and $288^{\circ }$ are achieved. A dual-line isolation structure is introduced to suppress mutual coupling between radiating units, ensuring cross-polarization levels (XPLs) better than −15.0 dB across the operation bands. Prototypes fabricated on a 160×160×1.5 mm^3^ substrate demonstrate measured $|S_{11}|<-10$ dB across 401–409 MHz and 2.34–2.53 GHz and stable omnidirectional patterns despite biasing circuitry perturbations. The compact form and robust dual-band, multi-polarization performance make the proposed antenna a promising candidate for implantable device wake-up signals and on-body data links in dense indoor environments.

## 1. Introduction

In recent years, wireless technologies have seen growing application in biomedical treatments, including remote monitoring, implantable medical device (IMD) communication, and intelligent health management [1,2,3]. Among these, body-centric wireless communication systems (BWCSs) have gained significant attention due to their ability to acquire and store patient physiological data in real time. A typical BWCS consists of an external data processing center (DPC) and multiple IMDs. To extend the operational lifespan of IMDs, these devices generally operate in two modes: sleep mode and wake-up mode. During wake-up mode, IMDs communicate with the DPC via the Medical Implant Communication Service (MICS) band (402–405 MHz), whereas in sleep mode, they operate in the Industrial, Scientific and Medical (ISM) 2.45 GHz band (2.4–2.48 GHz) solely to receive wake-up signals, thereby minimizing power consumption [4,5]. Consequently, both the antennas in IMDs and DPC need to support dual-band operation in the MICS and ISM bands to ensure system stability and efficiency. In addition, a dual-band DPC antenna can utilize the MICS and ISM bands separately to establish communication with implantable and on-body devices, enhancing system flexibility and robustness.

**Figure 1:**
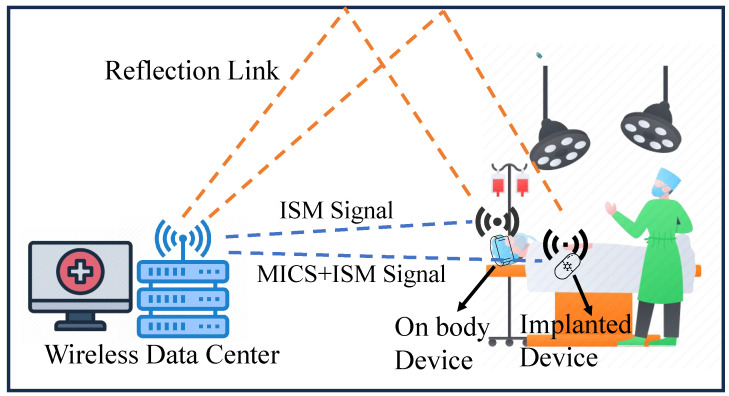
Schematic diagram of a BWCS with dual-frequency operation.

However, polarization mismatch is a major challenge in BWCSs. Since IMDs are implanted in unpredictable orientations within the body, and communication typically occurs in complex indoor environments [6], fixed single-polarized antennas at the DPC often suffer from polarization misalignment and multipath fading, as illustrated in Figure 1. These issues may degrade link quality significantly. To address this issue, researchers have proposed multi-linear polarization reconfigurable (MLPR) antennas, which dynamically switch LP states to align better with the IMD orientation and reduce signal fading [7,8,9,10]. For instance, in [7], a four LP reconfigurable antenna is designed for BWCS operation in the ISM 2.45 GHz band using a half-wavelength multi-dipole structure. Experimental results demonstrated its effectiveness in improving signal quality. Another study [9] employs an L-probe fed reconfigurable design to realize a four LP reconfigurable antenna supporting communication from 2.33 to 2.78 GHz. However, these antennas are not designed to operate in the MICS band. Simply scaling such structures to MICS frequencies would require large physical dimensions—for example, a diameter of about 370 mm at 403 MHz—which is not suitable for compact integration. Some studies have designed broadband MLPR antennas [11,12,13,14,15], such as [11], which covers 2.3–4.0 GHz by optimizing dipole structures. But due to the large frequency gap between the MICS and ISM 2.45 GHz bands, these broadband designs cannot effectively cover both bands simultaneously. Moreover, most of these antennas lack a compact design, resulting in large dimensions when designed to operate in the MICS band, limiting their applicability in integrated systems. Other efforts have aimed to enhance the multi-frequency capability of polarization-reconfigurable antennas [16,17,18,19]. For example, in [16], shorting vias are loaded along the patch edges, and a combination of PIN diodes and varactor diodes is employed. The ON/OFF states of PIN diodes enabled three LP states reconfiguration, while the capacitance variation of varactor diodes facilitated frequency tuning. The authors of [18] divide a circular patch into multiple sectors, integrating PIN diodes to control the radiation sector’s orientation and number, thereby achieving polarization switching and frequency agility. However, these designs only allow for continuous frequency tuning within a certain range and fail to simultaneously cover the widely separated MICS and ISM bands.

Another major difficulty lies in simultaneously supporting both bands within a compact antenna structure. Antennas operating in the MICS band inherently require larger sizes compared to those working at 2.45 GHz. Several studies have explored dual-band antenna designs for IMDs [20,21,22,23,24,25], but the radiation efficiency achieved by these antennas is usually low, making it difficult to be used as base station antennas for signal reception in DPC systems. Only a few studies have reported dual-band antennas with a significant frequency difference between the operating bands. For instance, [26] utilized two stacked magnetoelectric (ME) dipoles to achieve dual-band coverage from 300 MHz to 400 MHz and 1.9 GHz to 2.3 GHz. But the structure had a diameter exceeding 600 mm. Another design reported in [27] proposed an omnidirectional antenna composed of two stacked centrally fed circular patches, enabling operation in both the MICS and ISM 2.45 GHz bands. The radiation of the MICS frequency band was achieved within a diameter of 150 mm by adding through holes on the patch. However, these designs only support single-polarization operation, making them inadequate for increasingly complex BWCS application environments. To the best of our knowledge, no existing MLPR antenna design for a BWCS base station supports dual-band operation in both the MICS and ISM bands.

In this paper, we propose a novel MLPR antenna capable of operating in both the MICS and ISM bands while maintaining a compact size. The proposed antenna uses a multi-branch radiator structure, where each branch includes an elliptical patch for MICS band radiation and two strip patches for ISM 2.45 GHz band operation. A spring-shaped connecting branch enables compact integration, while an RF inductor is placed between the high-frequency and low-frequency sections to reduce mutual interference. Five such radiation units are symmetrically arranged around a central feed point. PIN diodes are used to control the connection between each unit and the feed. By switching these diodes on or off, the antenna can dynamically select among five different LP orientations. An isolation structure is implemented between adjacent units to suppress coupling and improve radiation performance. Finally, to verify the validity of the proposed antenna structure, we fabricated and measured two antenna prototypes. The results demonstrated that the proposed antenna could operate in the desired dual-frequency and five-LP states.

## 2. Antenna Design and Analysis

In this section, a configuration of the proposed dual-band MLPR antenna with five-LP reconfigurability is introduced. Subsequently, detailed multi-polarization and dual-frequency working mechanisms are described. Then, the effect of key parameters on the performance of the antenna is analyzed. The role of isolating branches employed in this antenna is also discussed.

### 2.1. Antenna Configuration

**Figure 2:**
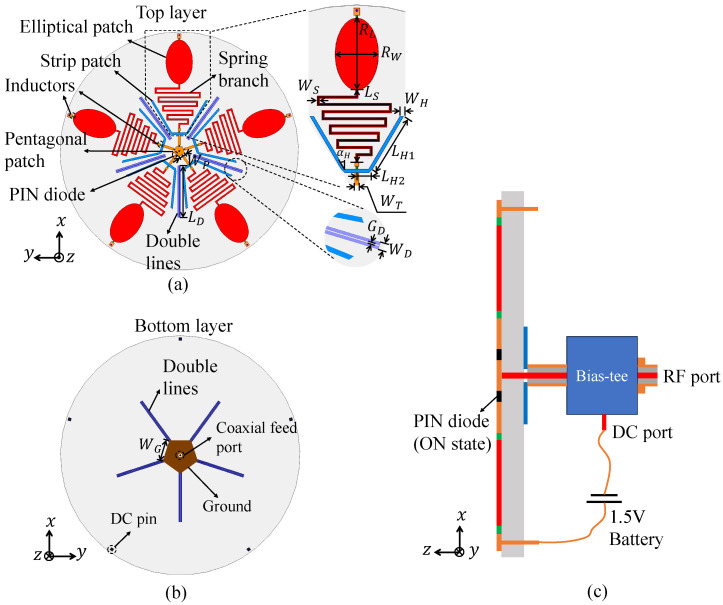
Schematic diagram of the proposed dual-band MLPR antenna. (**a**) Top layer of the antenna. (**b**) Bottom layer of the antenna. (**c**) Schematic diagram of DC bias circuit.

Figure 2 illustrates the configuration of the proposed dual-band MLPR antenna. The antenna is printed on a double-sided dielectric substrate with a relative permittivity ($\varepsilon_{r}$) of 3.55, a thickness of 1.5 mm, and a radius of 80 mm. It mainly consists of five multi-branch radiating units, arranged in a rotationally symmetrical manner with an interval of $72^{\circ }$, as shown in Figure 2a. Each radiating unit comprises an elliptical patch for low-frequency radiation and a pair of strip patches for high-frequency radiation, both printed on the top layer of the substrate. To accommodate the significant frequency gap between the MICS and ISM bands while maintaining compact integration, a curved spring branch is introduced to extend the current path of the elliptical patch, thereby reducing its resonance frequency within a constrained size. A central pentagonal feed patch with side length $W_{P}$ is printed on the top surface of the substrate, surrounded by the five radiating elements. The elliptical patch has dimensions of $R_{L}$ (length) and $R_{W}$ (width), while the spring branch has a total length of $L_{S}$ and a width of $W_{S}$. A DC pad is placed at the end of each elliptical patch. The two strip patches, serving as high-frequency radiating branches, are symmetrically positioned on either side of the spring branch. Each strip patch consists of an oblique branch and a transverse branch, with the oblique branch having a length $L_{H1}$ and an inclination angle $\alpha_{H}$, and the transverse branch having a length $L_{H2}$. The width of the strip patches is denoted as $W_{H}$. A chamfer is applied at the junction between the oblique and transverse branches to improve impedance matching. Each radiating unit is connected to the pentagonal feed patch via a microstrip line of length $L_{T}$ and width $W_{T}$. To suppress mutual coupling between adjacent radiating elements and improve radiation characteristics, a dual-line isolation structure with a slot is implemented between elements. This structure—characterized by length $L_{D}$, width $W_{D}$, and slot width $G_{D}$—is printed on both sides of the substrate. As shown in Figure 2b, the bottom layer also features a pentagonal ground patch with side length $W_{G}$, serving as the antenna’s ground plane. The proposed antenna is fed by a 50Ω coaxial probe, with its inner conductor connected to the central pentagonal feed patch and the outer conductor grounded to the ground.

To achieve polarization reconfigurability, PIN diodes are soldered between the pentagonal feed patch and each feedline. By selectively switching the PIN diodes, different radiating units can be activated, enabling polarization switching. In this work, to supply the required DC bias for the PIN diodes, we adopted a Bias-tee and a 1.5 V battery at the antenna feed end, as illustrated in Figure 2c. It should be pointed out that the Bias-tee and the battery are external devices that provide DC bias for the antenna. This approach is widely used and effective for the design and verification of reconfigurable antennas. For the proposed antenna, the negative terminal of the battery is connected to the DC port of the Bias-tee, and then connected to the negative terminals of all PIN diodes through the inner conductor of the coaxial probe and the pentagonal feed patch. The positive terminal of the battery is connected to a DC pin, which can be connected to the anode of the corresponding PIN diode through the corresponding DC pad and the metal patch printed on the top layer of the substrate, so that the diode is in the ON state.

In order to isolate the DC bias from the antenna radiation structure while maintaining the continuity of the DC current, two different high frequency inductors are incorporated into each radiating unit. Inductor 1 is positioned in the gap between the DC pad and the elliptical patch to isolate the RF current in the MICS band from the DC bias circuit. Inductor 2 is placed in the gap between the spring branch and the feed line, isolating the RF current in the ISM band from the low-frequency monopole and the DC bias circuit. Key parameter values of the proposed antenna are listed in Table 1.

### 2.2. Working Mechanism

The proposed antenna supports five LP reconfigurability by switching PIN diodes. For a specific polarization state, such as *x*-axis polarization, the PIN diode located along the *x*-axis is switched on by connecting the positive terminal of the battery to the corresponding DC pin. Thus, the the corresponding radiating unit is activated while the others are disconnected. This configuration forms a dual-band monopole antenna oriented along the *x*-axis, achieving *x*-axis polarization. By connecting the positive terminal of the battery to different DC pins, different corresponding PIN diodes can be turned on, achieving the switching of five LP states.

**Figure 3:**
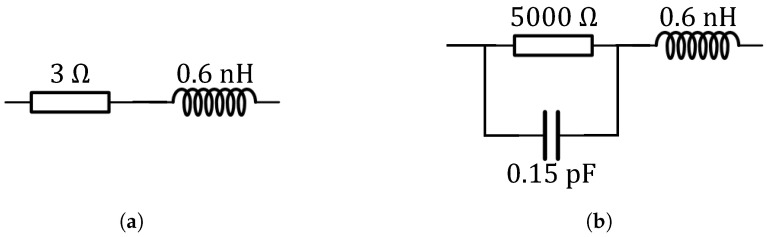
Equivalent circuit model of the adopted PIN diode. (**a**) ON state, (**b**) OFF state.

The PIN diode used in this design is the Infineon Bar50-02V, which operates within a frequency range of 10 MHz to 6 GHz. According to its datasheet in [28], the equivalent circuit model of the adopted PIN diodes are illustrated in Figure 3. In the ON state, it can be approximated as a 3Ω resistor in series with a 0.6 nH inductor, whereas in the OFF state, it can be modeled as a 5000Ω resistor in parallel with a capacitor with a capacitance value of $C_{T}$, both in series with a 0.6 nH inductor. The capacitance $C_{T}$ varies with frequency—approximately 0.17 pF in the MICS band and 0.15 pF in the ISM band. The two isolation inductors used are manufactured by Fenghua Advanced Technology. Inductor 1 (model VHF160808HR18JT) exhibits high impedance to RF currents around 400 MHz while maintaining DC continuity. Inductor 2 (model VHF160808H15NJH) provides high impedance around 2.45 GHz while acting as a 15 nH inductor for MICS band RF currents and DC signals. Detailed data for the above high-frequency inductors can be obtained from [29]. A 1.5 V battery supplies the necessary conduction current through a Bias-tee and DC pins.

**Figure 4:**
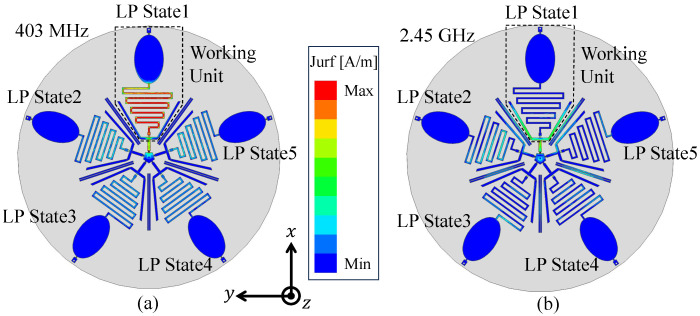
Current distribution of the antenna at different frequencies under $0^{\circ }$ LP. (**a**) 403 MHz, (**b**) 2.45 GHz.

The antenna’s radiating units operate as monopoles at different frequency bands due to the varied dimensions of their branches, enabling dual-band radiation in both the MICS and ISM 2.45 GHz bands. To verify this, surface current distributions were analyzed using the High Frequency Structure Simulator (HFSS). Figure 4a presents the surface current distribution when the antenna operates in the *x*-axis polarization state, excited by an RF signal at 403 MHz. It can be observed that the curved spring branch and elliptical patch create an extended current path, satisfying the typical quarter-wavelength requirement for monopole resonance. Figure 4b shows the surface current distribution of the antenna at 2.45 GHz, and it can be seen that most of the current is concentrated on the two strip patches. Inductor 2 effectively isolates these patches from the low-frequency monopole, concentrating the radiating currents. Each strip patch forms a monopole in the ISM 2.45 GHz band to achieve effective radiation.

### 2.3. Parameter Study

Each radiating unit of the proposed dual-band MLPR antenna comprises multiple branches that form both low-frequency and high-frequency monopoles. Although the underlying principle is straightforward, the presence of numerous structural parameters necessitates a simulation-driven design process to determine optimal values.

**Figure 5:**
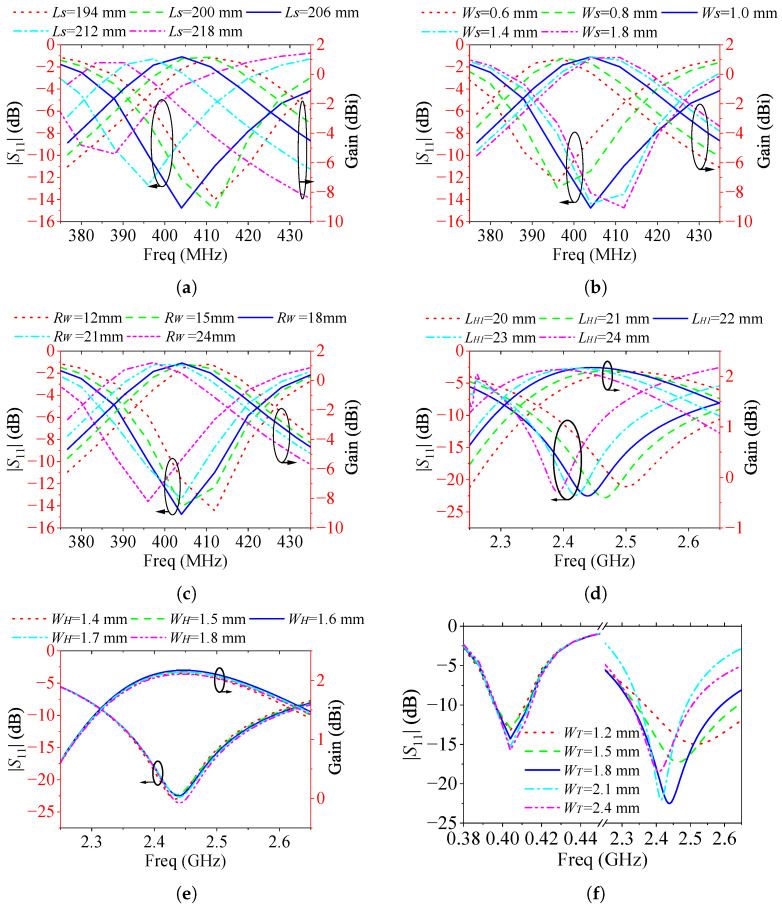
The effect of different main parameters on the performance of the antenna in $0^{\circ }$ LP. (**a**) $L_{S}$, (**b**) $W_{S}$, (**c**) $R_{W}$, (**d**) $L_{W}$, (**e**) $W_{H}$, (**f**) $W_{T}$.

The low-frequency monopole consists of a spring branch and an elliptical patch. Due to the compact geometry of the spring branch, increasing its length $L_{S}$ can reduce the required length of the elliptical patch $R_{L}$ while maintaining the total electrical length of the low-frequency monopole. This enables a reduction in the monopole’s overall size. However, a trade-off must be addressed: the elliptical patch’s size is positively correlated with the effective radiating aperture area. If $R_{L}$ becomes too small, the antenna gain may deteriorate. Furthermore, the dimensions of the spring branch affect the transverse size of the monopole. An excessively large transverse footprint can hinder the rotationally symmetric arrangement of multiple elements due to spatial constraints. To balance these considerations, the size of each multi-branch radiating element is confined within a sector of radius approximately 80 mm and angular width $72^{\circ }$. To avoid significant gain degradation in the MICS band, the elliptical patch length is fixed at $R_{L}=30$ mm. Consequently, the overall length of the low-frequency monopole is primarily governed by the spring branch length $L_{S}$. Figure 5a illustrates the simulated $|S_{11}|$ and gain curves over 380–430 MHz for varying $L_{S}=\left[194,200,206,212,218\right]$ mm, with fixed values $W_{S}=1.0$ mm and $R_{S}=18$ mm. The results show that for $L_{S}=200$ mm and 206 mm, impedance matching better than −10 dB is achieved across the MICS band, along with a relatively stable gain of approximately 1.52 dBi. Based on this trade-off, $L_{S}=206$ mm is selected. The spring branch line width $W_{S}$ also influences low-frequency performance due to its connection to the feedline and the elliptical patch. Figure 5b presents the $|S_{11}|$ and gain curves for different $W_{S}$ values. When $W_{S}=1.0$ mm, the working band of the antenna covers 395–414 MHz, offering good impedance performance.

For the elliptical patch, increasing the width $R_{S}$ generally smoothens the impedance curve, aiding bandwidth enhancement. However, based on antenna quality factor (*Q*) theory, increased bandwidth often comes at the cost of reduced gain. Therefore, a suitable $R_{S}$ must balance bandwidth and gain performance. Figure 5c shows that when $R_{S}=18$ mm, the antenna exhibits the best combined impedance and gain characteristics in the MICS band. The high-frequency monopole is formed by transverse and oblique branches. The transverse branch allows the high-frequency structure to be distributed along the edge of the low-frequency monopole, achieving structural integration. In this design, the transverse branch length $L_{H1}$ is set to 6.5 mm, and the tilt angle $\alpha_{H}$ is fixed at $30^{\circ }$. The primary parameter influencing high-frequency monopole performance is the oblique branch length $L_{H2}$. Figure 5d presents the $|S_{11}|$ and gain curves over 2.3–2.6 GHz for $L_{H2}=\left[20,\;21,\;22,\;23,\;24\right]$ mm. It can be observed that for $L_{H2}=21$ mm and 22 mm, the antenna achieves wide coverage of 2.38–2.58 GHz and 2.35–2.56 GHz, respectively. Therefore, $L_{H2}=22$ mm is selected for optimal performance. The width $W_{H}$ of the high-frequency monopole also affects the impedance characteristics. Figure 5e shows that when $W_{H}=1.6$ mm, the antenna demonstrates good impedance matching and stable gain across the desired ISM 2.45 GHz band.

In addition to the above parameters, the feedline width $W_{T}$ impacts impedance matching at both operating bands. Figure 5f presents $|S_{11}|$ curves for $W_{T}=\left[1.2,\;1.5,\;1.8,\;2.1,\;2.4\right]$ mm. The MICS band is relatively insensitive to $W_{T}$, but in the ISM band, good matching is achieved when $W_{T}=1.5$ mm and 1.8 mm. Considering practical fabrication requirements, a wider feedline is preferred to facilitate soldering of PIN diodes and inductors. Therefore, $W_{T}=1.8$ mm is chosen.

### 2.4. Effect of the Isolating Branch

**Figure 6:**
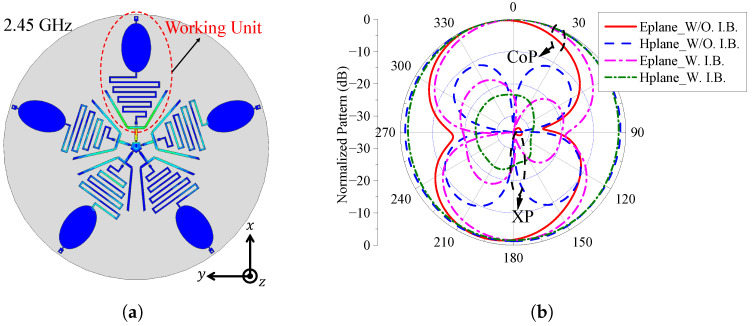
(**a**) The current distribution of the antenna in $0^{\circ }$ LP at 2.45 GHz without isolating branches. (**b**) Simulated pattern of the antenna in $0^{\circ }$ LP at 2.45 GHz with or without isolating branches. W/O. I.B. represents the antenna without the isolation branches; W. I.B. represents the antenna with the isolation branches; CoP represents co-polarization; XP represents cross-polarization.

Due to the compact layout of the proposed dual-band MLPR antenna, the active radiating branches are susceptible to interference from adjacent inactive branches. During the simulation-based design process, it was observed that in the absence of isolating branches, strong electromagnetic coupling occurs between active and neighboring inactive units, particularly in the ISM band. As illustrated in Figure 6a, such coupling leads to elevated cross-polarization levels (XPLs) in certain directions, thereby degrading the overall radiation purity, as shown in Figure 6b. To address this issue and achieve low XPL across all directions, we introduce isolating branches between adjacent radiating elements. Inspired by the approach in [30], these isolating branches adopt a dual-line structure that forms additional coupling paths. The induced currents in these paths are designed to be out-of-phase with the original near-field coupling currents, leading to effective cancellation and suppression of mutual coupling effects. Figure 6b also presents the radiation pattern of the antenna at 2.45 GHz with the isolating branches included. It is evident that, compared to the configuration without isolation structures, the XPL is significantly reduced, demonstrating the effectiveness of the proposed decoupling strategy.

## 3. Fabrication and Measurement Results

**Figure 7:**
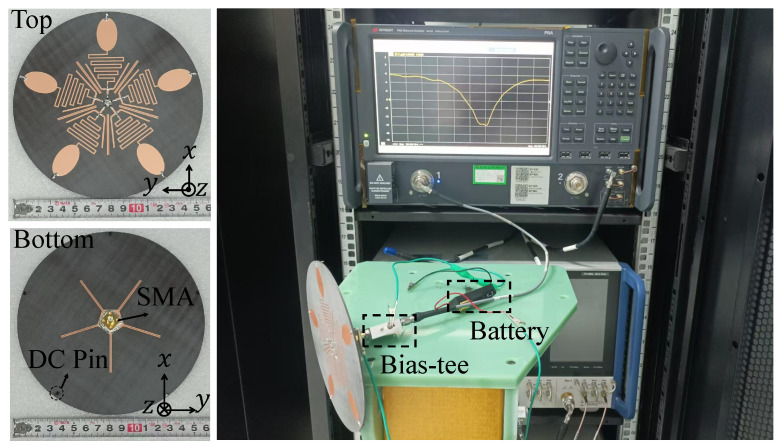
Photos of the fabricated antenna Prototype 1 and on-site photo taken during the measurement of $|S_{11}|$.

To further validate the proposed antenna structure, a prototype—referred to as Prototype 1—is fabricated and measured, as shown in Figure 7. The reflection coefficient characteristics are evaluated using a Keysight N5224B Vector Network Analyzer (VNA), with a measuring frequency range of 10 MHz to 43.5 GHz. Since the antenna Prototype 1 operates in a dual-frequency band with a large span, in order to make the measurement results accurate, we calibrated the VNA and measured the Prototype 1 in two frequency bands. Mechanical calibration parts were used for VNA calibration. According to the intelligent calibration program of VNA, once the mechanical calibration parts are connected and calibrated in sequence, the calibration can be completed. In this work, the proposed antenna usually does not come into direct contact with the human body, so the antenna prototype is measured as its condition in free space.

**Figure 8:**
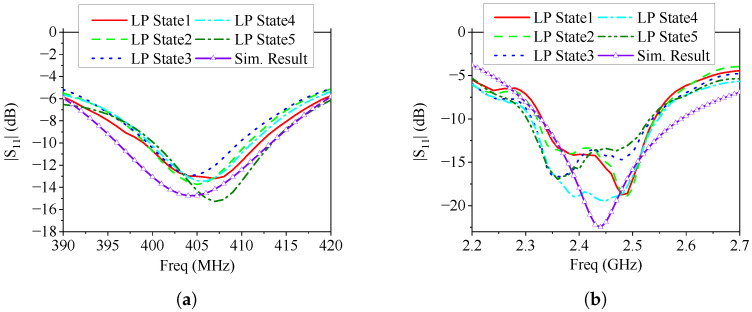
Measured $|S_{11}|$s of the fabricated antenna Prototype 1 in free space under different polarization states, (**a**) 390–420 MHz; (**b**) 2.20–2.70 GHz.

The simulated and measured $|S_{11}|$ curves are presented in Figure 8. Due to the rotational symmetry of the proposed antenna, this paper only presents the simulation results of the antenna in one polarization state for comparison. The measured $|S_{11}|$ values under five different polarization states exhibit good consistency. Although there are some differences between the measured $|S_{11}|$ and the simulated one due to manufacturing and welding errors, the measured results confirm that the antenna’s overlapping operating bands cover 401–409 MHz and 2.34–2.53 GHz, effectively encompassing the MICS and ISM 2.45 GHz frequency bands.

**Figure 9:**
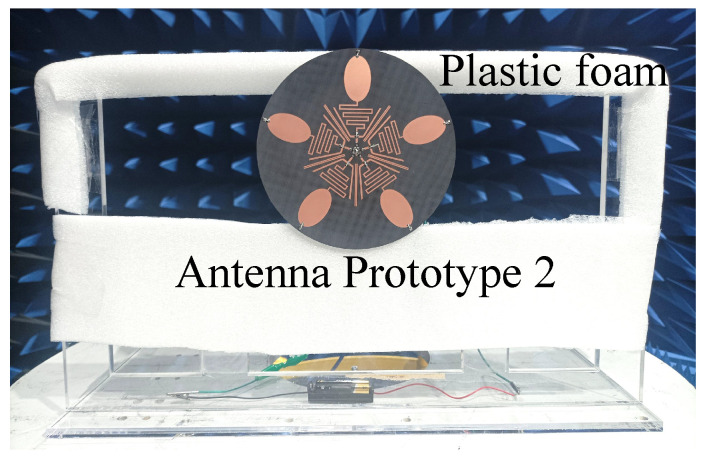
Photos of the fabricated antenna Prototype 2 and on-site photo taken during the measurement of radiation patterns. Key parameters value of the fabricated antenna Prototype 2: $R_{L}=25.0$ mm, $R_{W}=15$ mm, $L_{S}=172.0$ mm, $W_{S}=1.0$ mm, $L_{H1}=22.0$ mm, $L_{H2}=6.5$ mm, $\alpha_{H}=30^{\circ }$, $W_{H}=1.6$ mm, $L_{T}=7.0$ mm, $W_{T}=1.8$ mm, $L_{D}=35.0$ mm, $W_{D}=1.9$ mm, $G_{D}=0.3$ mm, $W_{P}=4.3$ mm, $W_{G}=14.5$ mm.

**Figure 10:**
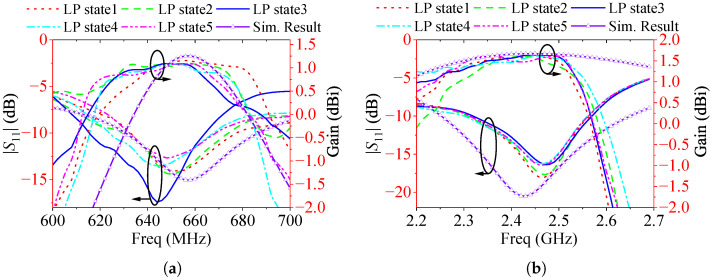
Measured $|S_{11}|$ and gain curves of the fabricated antenna Prototype 2 under two different frequency bands, (**a**) 600–700 MHz; (**b**) 2.20–2.70 GHz.

**Figure 11:**
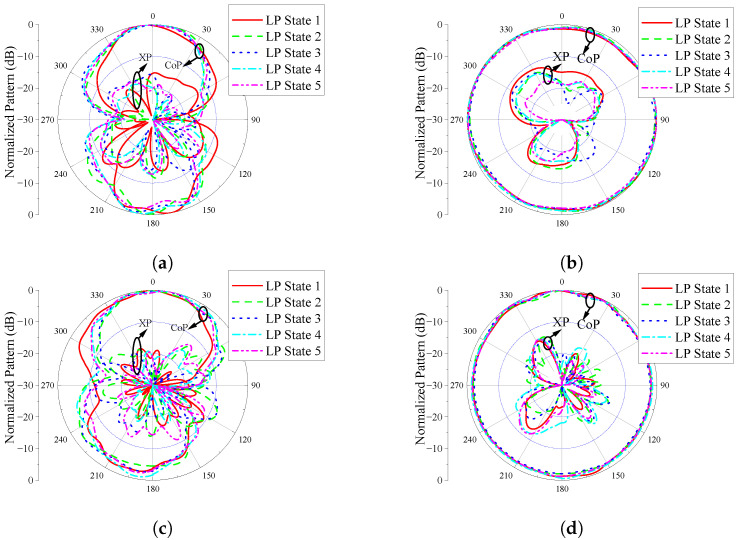
Measured patterns of the fabricated antenna Prototype 2 under different polarization states, (**a**,**b**) Eplane and Hplane patterns at 650 MHz; (**c**,**d**) Eplane and Hplane patterns at 2.45 GHz.

Since the microwave anechoic chamber in our laboratory does not support measurements below 600 MHz, an additional prototype—referred to as Prototype 2—was fabricated to evaluate the radiation pattern performance of the proposed design. This prototype is designed to operate at 650 MHz and 2.45 GHz, as illustrated in Figure 9. Its structure is identical to that shown in Figure 2, except for the scaled dimensions. Based on the parameter study in the previous section, we obtained the size parameters of Prototype 2, mainly adjusting the length of the spring branch ($L_{S}$) and the length of the elliptical patch ($R_{L}$). The other key parameters are also optimized to enable the antenna Prototype 2 to operate at the two desired frequencies. The detailed dimensions are provided in the caption of Figure 9. When measuring the radiation patterns, antenna Prototype 2 is fixed on the bracket with plastic foam with a dielectric constant close to 1 to obtain a better measurement environment. The measured $|S_{11}|$ and gain curves of Prototype 2 are shown in Figure 10. The measured $|S_{11}|$ has a certain offset compared to the simulated $|S_{11}|$, but overall it is still in good agreement. The results indicate that the antenna resonates near 650 MHz and 2.45 GHz, with measured operating bands spanning 634–663 MHz and 2.33–2.55 GHz. There is a certain difference between the measured and simulated gain curves, mainly due to the distortion of the antenna pattern caused by the performance of the actual RF inductor varying with frequency. The measured gain of Prototype 2 ranges from 0.67 to 1.21 dBi in the low-frequency band, and from 0.81 to 1.66 dBi in the high-frequency band. Although there are some differences between the measured and simulated gain curves, the gain of the antenna is stable within the concerned working frequency band. The measured radiation patterns of Prototype 2 are presented in Figure 11. As can be seen, the E- and H-patterns of the antenna maintain good consistency under different polarization states. The pattern shape of the antenna is consistent with that of a monopole antenna. And the overall XPL is better than −15.0 dB. Although some ripples occur on the radiation patterns, the antenna maintains an omnidirectional radiation performance in both frequency bands.

In summary, Prototype 1 successfully operates in the MICS and ISM 2.45 GHz bands, while Prototype 2 operates at both 650 MHz and 2.45 GHz. These measurement results validate the proposed antenna structure’s capability to achieve compact, dual-band operation with multi-linear polarization functionality.

## 4. Discussion

### 4.1. Comparison of the Radiation Pattern of Prototype 1 and Prototype 2

**Figure 12:**
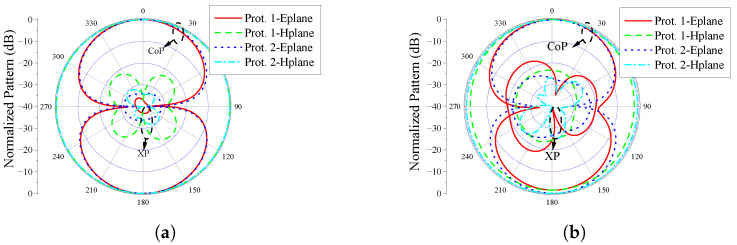
Comparison of simulated patterns of Prototype 1 and Prototype 2. (**a**) Prototype 1 at 403 MHz and Prototype 2 at 650 MHz; (**b**) Both prototypes are at 2.45 GHz.

In this work, due to the limitations of the measurement conditions, we did not directly measure the pattern of Prototype 1 operating in the MICS and ISM 2.45 GHz frequency band. Instead, an additional Prototype 2 with the same structure but operating at 650 MHz and 2.45 GHz was designed to verify the pattern performance of the proposed antenna. To further illustrate that the pattern performance implemented by Prototype 1 is similar to that of Prototype 2, we present a comparison of the simulated patterns of the two in Figure 12. For convenience of comparison, only the patterns of Prototype 1 and Prototype 2 when they are in polarization state 1 are shown in the figure. It can be seen that the two match very well. From this, combined with the pattern measurement results of Prototype 2, it can be inferred that the pattern of the fabricated Prototype 1 should also meet the expectations. Essentially, the radiation principles of the two antennas are identical, both directly forming compact monopole antennas in two frequency bands. Hence, the radiation performance of the two is similar in theory.

### 4.2. Performance Comparison with Existing Related Antennas

Table 2 summarizes the statistical performance data of the proposed dual-band MLPR antenna presented in this paper, along with several representative designs reported in related studies. Compared with works such as [7,8,9,10,18], which operate solely within the 2–3 GHz band, our design supports dual-band operation covering both the MICS band and the ISM 2.45 GHz band, making it more versatile for BWCS applications. In terms of polarization diversity, the proposed antenna achieves five distinct LP states, outperforming designs with only one to four LPs (e.g., [7,9,26]) and offering a more compact and simpler structure compared to the 16-LP solution in [18]. Moreover, the proposed antenna exhibits a miniaturized volume of only $0.21\times 0.21\times 0.002{\lambda_{L}}^{3}$ and $1.30\times 1.30\times 0.012{\lambda_{H}}^{3}$ at the two operating bands, representing one of the smallest form factors among the listed designs, particularly at the low-frequency MICS band. In terms of radiation efficiency, our design achieves 64.8% at 400 MHz and 83.2% at 2.45 GHz, which is competitive with or better than most other dual-band or wideband designs, while maintaining compact dimensions and reliable polarization performance. These results demonstrate that the proposed antenna offers an excellent balance between compact size, dual-band operation, efficient radiation, and multiple polarization states, making it a strong candidate for modern wireless body-area communication systems.

### 4.3. Performance of the Proposed Antenna near a Human Head

**Figure 13:**
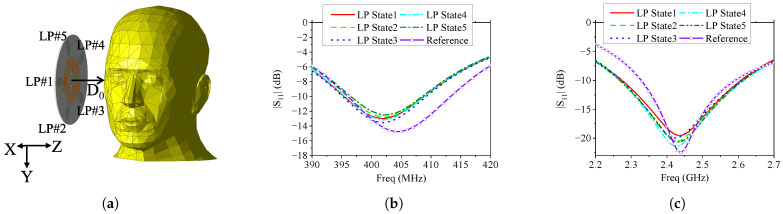
(**a**) Simulation model of the proposed antenna placed beside a human head. (**b**) Simulated $|S_{11}|$ curves in MICS band. (**c**) Simulated $|S_{11}|$ curves in ISM 2.45 GHz band. The distance between the antenna surface and the human head surface is 15 cm. “Reference” indicates the result of the antenna in free space.

In practical BWCS scenarios, the proposed antenna may be deployed around or near a human body for wireless data acquisition. Hence, it is necessary to evaluate the impact of human tissue on antenna performance and the specific absorption rate (SAR) to ensure human safety. To investigate this, a simplified simulation was conducted by placing a human head at a typical interaction distance of approximately 15.0 cm from the antenna surface. The setup is illustrated in Figure 13a, where the human head model is a model from the HFSS built-in library. In this case, we obtained the $|S_{11}|$ of the antenna in each polarization state and the SAR inside the human head through simulation.

Figure 13b,c show the simulated $|S_{11}|$ curves of the antenna in the MICS and ISM bands, respectively. The $|S_{11}|$ of the antenna in free space (Reference) are also given in the figure for comparison. It can be observed that the antenna maintains $|S_{11}|<-10$ dB in both frequency bands, even in the presence of the human head, indicating robust impedance matching. In the MICS band, a slight downward shift in resonance frequency (1–2 MHz) is noted, accompanied by a marginal degradation in return loss. This is attributed to the high dielectric constant and conductive nature of biological tissues, which influence the near-field reactive impedance of the low-frequency monopole structure. In the ISM 2.45 GHz band, the impact is slightly smaller, with the resonance frequency shifting down by approximately 5 MHz and a small increase in $|S_{11}|$ values for certain frequency points. Nonetheless, the antenna still operates effectively across the full ISM 2.45 GHz band. These results demonstrate that the proposed MLPR antenna exhibits stable impedance performance in realistic body-centric scenarios.

**Figure 14:**
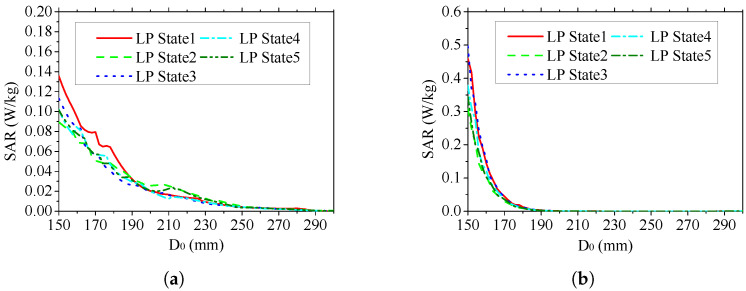
Simulated SAR of the proposed antenna placed beside a human head. (**a**) Simulated SAR curve of the antenna at 403 MHz. (**b**) Simulated SAR curve of the antenna at 2.45 GHz. Variable $D_{0}$ represents the distance from the observation point to the center of the antenna surface.

In Figure 14, we show the SAR of the human head obtained by simulation with an input power of 1 W. The variable $D_{0}$ represents the distance from the center of the antenna surface to the observation point. Since the distance between the antenna surface and the human head surface is set to 150 mm, the range of $D_{0}$ is given as 150–300 mm. Figure 14a,b show the SAR curves at 403 MHz and 2.45 GHz, respectively. It can be seen that the SAR value gradually decreases with increase in distance. In both frequency bands, the maximum SAR value does not exceed 0.5 W/kg. In this simulation case, the SAR of the antenna meets the general safety requirements (less than 1.6 W/kg).

## 5. Conclusions

This work has introduced and validated a compact dual-band MLPR antenna capable of dynamically switching among five LPs to mitigate polarization mismatch and multipath fading in BWCSs. The innovative integration of a spring branch with an elliptical patch realizes low-frequency resonance within a constrained footprint, while two strip patches ensure high-frequency operation. A dual-line isolating branch effectively suppresses inter-element coupling, preserving radiation purity. Measurement results from two fabricated prototypes confirm wide coverage of the MICS and ISM 2.45 GHz bands with consistent impedance matching, stable gains, and XPLs exceeding design goals. Compared to existing solutions, the antenna uniquely combines dual-band functionality with five-state polarization agility in a compact layout. Furthermore, this paper also investigates the radiation performance of the antenna when it is near a human head, demonstrating good robustness and radiation safety. Future work will explore expansion of the antenna bandwidth, the performance of antennas under the influence of different human tissues, and the improvement in working performance in a real-world medical equipment environment.

## Figures and Tables

**Table 1:** Main parameters of the proposed dual-band MLPR antenna and their values.

Parameter	Description	Value	Parameter	Description	Value
$R_{L}$	Length of the elliptical patch	30.0 mm	$R_{W}$	Width of the elliptical patch	18.0 mm
$L_{S}$	Length of the spring branch	206.0 mm	$W_{S}$	Width of the spring branch	1.0 mm
$L_{H1}$	Length of the oblique branch	22.0 mm	$L_{H2}$	Length of the traverse branch	6.5 mm
$\alpha_{H}$	Obliquity of the oblique branch	$30^{\circ }$	$W_{H}$	Width of the strip patch	1.6 mm
$L_{T}$	Length of the feedline	7.0 mm	$W_{T}$	Width of the feedline	1.8 mm
$L_{D}$	Length of the doubleline	35.0 mm	$W_{D}$	Width of the doubleline	1.9 mm
$G_{D}$	Gap width of the doubleline	0.3 mm	$W_{P}$	Width of the pentagonal feed patch	4.3 mm
$W_{G}$	Width of the pentagonal ground	14.5 mm			

**Table 2:** Performance comparison with the antennas reported in some related studies.

References	Antenna Type	Achieved Polarization	Operating Frequency Band	Radiation Efficient	Antenna Dimensions
[7]	Multi-dipole antenna	Four LPs	2.2−3.1 GHz	80–90%	$0.88\times 0.88\times 0.265{\lambda_{C}}^{3}$
[8]	Patch antenna	Six LPs	2.4 GHz	46%	$1.04\times 1.04\times 0.024{\lambda_{C}}^{3}$
[9]	L-probe antenna	Four LPs	2.325−2.775 GHz	/	$0.68\times 0.68\times 0.14{\lambda_{C}}^{3}$
[10]	Patch antenna	Four LPs	2.33−2.50 GHz	82.9–84.7%	$0.56\times 0.56\times 0.07{\lambda_{C}}^{3}$
[16]	Patch antenna	Three LPs	1.35−1.90 GHz	/	$0.38\times 0.38\times 0.017{\lambda_{C}}^{3}$
[18]	Patch antenna	Sixteen LPs	1.76−2.64 GHz	66.8%	$0.70\times 0.70\times 0.10{\lambda_{C}}^{3}$
[26]	ME dipole	Single LP	300−400 MHz 1.9−2.3 GHz	/	$1.75\times 1.75\times 0.28{\lambda_{L}}^{3}$ $10.5\times 10.5\times 1.68{\lambda_{H}}^{3}$
[27]	Patch antenna	Single LP	395−405 MHz 2.4−2.48 GHz	54.8% 88.6%	$0.20\times 0.20\times 0.005{\lambda_{L}}^{3}$ $1.23\times 1.23\times 0.034{\lambda_{H}}^{3}$
Our work	Multi-branch monopole antenna	Five LPs	401−409 MHz 2.34−2.53 GHz	64.8% 83.2%	$0.21\times 0.21\times 0.002{\lambda_{L}}^{3}$ $1.30\times 1.30\times 0.012{\lambda_{H}}^{3}$

$\lambda_{L}$ and $\lambda_{H}$ are, respectively, the low-frequency and high-frequency operating points of the dual-frequency antennas listed in the table, and $\lambda_{C}$ is the center frequency point of the antennas operating in a continuous frequency band listed in the table.

## Data Availability

Data are contained within the article.

## Abbreviations

The following abbreviations are used in this manuscript:

MLPR	Multi-linear polarization reconfigurable antenna
XPL	Cross-polarization level
PIN	Positive-intrinsic-negative
IMD	Implantable medical device
BWCS	Body-centric wireless communication systems
DPC	Data processing center
MICS	Medical implant communication service
ISM	Industrial, Scientific and Medical
RF	Radio frequency
DC	Direct current
HFSS	High-frequency structure simulator
